# Neurodegeneration, Mitochondria, and Antibiotics

**DOI:** 10.3390/metabo13030416

**Published:** 2023-03-12

**Authors:** Juan M. Suárez-Rivero, Juan López-Pérez, Inés Muela-Zarzuela, Carmen Pastor-Maldonado, Paula Cilleros-Holgado, David Gómez-Fernández, Mónica Álvarez-Córdoba, Manuel Munuera-Cabeza, Marta Talaverón-Rey, Suleva Povea-Cabello, Alejandra Suárez-Carrillo, Rocío Piñero-Pérez, Diana Reche-López, José M. Romero-Domínguez, José Antonio Sánchez-Alcázar

**Affiliations:** 1 Institute for Biomedical Researching and Innovation of Cádiz (INiBICA) University Hospital Puerta del Mar, 11009 Cádiz, Spain; 2 Department of Molecular Biology Interfaculty Institute for Cell Biology, University of Tuebingen, D-72076 Tuebingen, Germany; 3 Andalusian Centre for Developmental Biology (CABD-CSIC-Pablo de Olavide-University), 41013 Sevilla, Spain

**Keywords:** neurodegeneration, mitochondria, antibiotics, neuroinflammation, neurodegenerative diseases

## Abstract

Neurodegenerative diseases are characterized by the progressive loss of neurons, synapses, dendrites, and myelin in the central and/or peripheral nervous system. Actual therapeutic options for patients are scarce and merely palliative. Although they affect millions of patients worldwide, the molecular mechanisms underlying these conditions remain unclear. Mitochondrial dysfunction is generally found in neurodegenerative diseases and is believed to be involved in the pathomechanisms of these disorders. Therefore, therapies aiming to improve mitochondrial function are promising approaches for neurodegeneration. Although mitochondrial-targeted treatments are limited, new research findings have unraveled the therapeutic potential of several groups of antibiotics. These drugs possess pleiotropic effects beyond their anti-microbial activity, such as anti-inflammatory or mitochondrial enhancer function. In this review, we will discuss the controversial use of antibiotics as potential therapies in neurodegenerative diseases.

## 1. Introduction

Neurodegenerative diseases are generally classified according to their clinical presentation, with movement, cognitive, and behavioral disorders being the most common. Diagnosis is generally difficult given the fact that patients normally present heterogeneous clinical features. Very often, neurodegenerative diseases are diagnosed post-mortem via neuropathological evaluation at autopsy. Nevertheless, there is currently great interest among the scientific community in the identification of biomarkers or specific genetic mutations that help clinicians anticipate the onset of these diseases to either treat them when still reversible or slow down their progression [1]. This is especially relevant given the fact that the characteristic protein abnormalities linked to these diseases are present in patients long before the clinical symptoms become noticeable [2,3].

Neurodegenerative diseases share a series of common traits such as proteotoxic stress, abnormalities in the autophagosomal/lysosomal and ubiquitin/proteosomal systems, lipid peroxidation, iron accumulation, neuroinflammation, oxidative stress, mitochondrial dysfunction, and eventually neuronal death. Among them, mitochondrial dysfunction has long been demonstrated as a prominent early pathological event of a variety of neurodegenerative diseases [4]. In fact, in no cell type is mitochondrial function more vital than in neurons. First of all, because their limited glycolytic activity causes them to rely primarily on oxidative phosphorylation (OXPHOS), second, because their long axons require energy-dependent transport of cell components over long distances, and third, because synaptic transmission is dependent on calcium signaling, which needs energy-dependent regulation [5]. One of the first signs hinting towards the involvement of mitochondria in neurodegenerative diseases was observed in Alzheimer’s disease (AD) patients, who presented reduced glucose metabolism in the brain with respect to healthy individuals [6]. This fact has been reported in many studies using 18-FDG positron emission tomography (PET) to determine the rate of metabolic activity in AD patients. Interestingly, according to this finding, the reduction of metabolic activity in AD patients correlated with their degree of cognitive impairment [6]. In addition, it has been demonstrated that AD patients present impaired oxygen consumption in the brain, further adding to the notion that bioenergetic dysfunction and mitochondrial impairment are common features of AD [7]. Indeed, impairment of several mitochondrial enzymes such as pyruvate dehydrogenase (PDH) and ketoglutarate dehydrogenase (KGDH) has been detected in AD patients. Among mitochondrial oxidative phosphorylation complexes, cytochrome c oxidase (complex IV) is the most affected. Its activity was found to be significantly reduced in the brain tissue of AD patients, which represented a general decrease in functionality of the electron transport chain although other mitochondrial proteins were not altered [8]. Moreover, mitophagy has been proposed as a key factor to consider in the pathogenesis of AD. In fact, an impairment of the mitophagy machinery has been reported in both human and biological models of AD [9]. In this line, stimulation of mitophagy has proven to be effective to improve neuropathological and clinical features in these models [9,10].

In addition to AD, there is compelling evidence to support the involvement of mitochondria in the pathogenesis of different neurodegenerative diseases, with their role being particularly relevant in Parkinson’s disease (PD). According to numerous studies, patients with this condition present a selective deficiency of respiratory chain complex I, which is most remarkable when enzymatic activity is measured in the substantia nigra (SN) [11,12]. The role of complex I in the pathogenesis of PD is supported by the finding that the administration of complex I inhibitors, namely 1-methyl-4-phenyl-1,2,5,6-tetrahydropyridine (MPTP) or rotenone, to both humans and animal models leads to the onset of parkinsonism and striatonigral degeneration [13,14,15]. Rotenone, in particular, not only triggers motility-related symptoms of PD such as hypokinesia, rigidity, and shaking but also leads to dopaminergic neuron degeneration and cytoplasmic aggregations of α-syn and ubiquitin in rats [16]. Furthermore, there seems to be a link between mitochondrial DNA (mtDNA) and the pathogenesis of PD. A high mtDNA deletion burden in the SN has been reported among PD patients [17], being further corroborated in a study evaluating DNA from SN individual neurons of patients and healthy donors [18]. Moreover, the amount of mtDNA is altered in PD patients [19] and most of the mutations that have been identified as causative of familial PD occur in genes that are directly involved in mitochondrial biology, the most prominent examples being Parkin and PINK1 [20,21]. Apart from Alzheimer’s and Parkinson’s diseases, mitochondrial perturbations have been reported in many other neurodegenerative diseases, among which amyotrophic lateral sclerosis (ALS), Huntington’s disease (HD), and neurodegeneration with brain iron accumulation (NBIA) disorders deserve special mention [22,23,24,25]. Nevertheless, a critical question that remains unanswered is whether mitochondrial dysfunction is indeed one of the main factors leading to neurodegeneration or just a collateral feature arising from alternative phenomena such as the accumulation of misfolded proteins, lipid peroxidation, iron accumulation, autophagy/mitophagy dysfunction, or cellular stress. All these instances could be part of a continuous and vicious cycle as has been recently proposed [26]. Therefore, targeting mitochondrial dysfunction is a promising therapeutic strategy for neurodegenerative pathologies. Recent studies have demonstrated that mitochondria-targeted antioxidants counteract the excessive ROS production associated with mitochondrial malfunction and present neuroprotective properties in cultured cells and a mouse model of ALS [27]. Another therapeutic strategy in this context is focused on targeting energy dysfunction in patients by supplementing them with creatine, an intracellular energy buffer in the brain and skeletal muscle. Creatine supplementation achieved outstanding results in vitro and in vivo and it has been evaluated in clinical trials for both PD and Huntington’s disease (HD). The outcome for PD was promising, with one study showing a reduction in disease progression of 50% after one year of treatment [28]. 

Other therapeutic approaches in neurodegenerative diseases include the treatment of calcium dysregulation, defects in mitochondrial fission and fusion, disrupted mitochondrial protein import, aberrant mitochondrial kinases, or dysfunctional mitochondrial protein quality control such as mitophagy, mitochondria-derived vesicles (MDV), or mitochondrial unfolded protein response (UPR^mt^). The UPR^mt^ is a mechanism aimed to preserve or repair damaged mitochondria. It is responsible for maintaining mitochondrial proteostasis through the activation of a transcriptional program in the nuclear DNA [29]. Although the molecular mechanism of UPR^mt^ in humans is not fully understood, it is gaining relevance in a variety of physiological processes such as aging, oxidative stress resistance, hematopoietic stem cell maintenance, glycolysis, antibacterial immunity, coenzyme Q biosynthesis, mitochondrial fission, and neurodegeneration [30,31]. Loss of mitochondrial proteostasis is the main factor inducing the UPR^mt^, especially when the accumulation of damaged proteins exceeds the protein-processing capacity of the mitochondrial chaperones and proteases [32]. Furthermore, mitochondrial function stressors and/or damage also promote UPR^mt^ induction. Examples of these are the inhibition of complex I by rotenone [33], bacterial toxins [34], knockdown of quality control proteins [35], or generation of excess ROS by paraquat [36].

Antibiotics are a group of molecules with the capacity of disrupting several bacterial mechanisms such as DNA, RNA, protein, and cell wall synthesis, thus promoting bacterial death [37]. Nevertheless, the alternative use of these drugs has proven to be very promising for the treatment of a wide range of conditions, ranging from cancer to neurodegenerative diseases or aging [38]. Due to their bacterial origin, and the fact that they conserve prokaryotic features such as the 55S or 60S ribosomes, mitochondria are exceptionally sensitive to antibiotics. However, it is precisely for this reason that treating patients with antibiotics for long time spans is an extremely controversial approach [38]. Thus, clinically relevant doses of several antibiotics cause mitochondrial dysfunction in mammalian cells [39]. Moreover, plant chloroplasts and mitochondria are also vulnerable to antibiotics that are released into the environment [40].

Furthermore, antibiotics can cause significant changes in gut microbiota and the development of bacterial antibiotic resistance that have both short- and long-term health consequences [41,42]. It is known that gut microbiota may affect the activity of the nervous system by producing various neurotransmitters and mediators as well as toxic substances and/or releasing pro-inflammatory cytokines, which can either cross the blood–brain barrier (BBB) or send a signal to the brain via the vagus nerve [43]. Some authors warned that gut microbial dysbiosis caused by antibiotics may lead to alterations in brain functions [44]; however, other studies showed that some antibiotics could be beneficial by reducing pro-inflammatory bacteria and optimizing drug absorption such as levodopa in PD [45,46]. 

**Figure 1:**
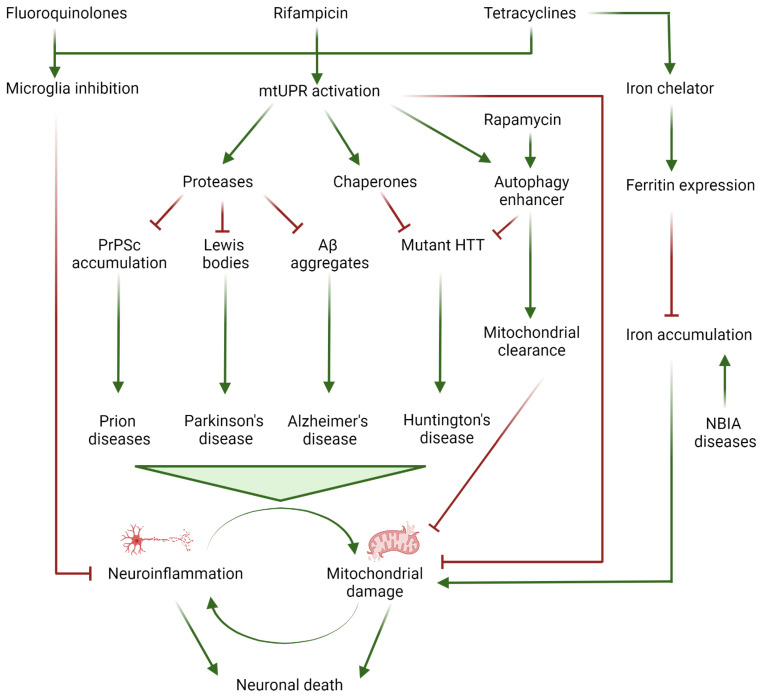
The proposed mechanisms of action of several antibiotics in neurodegenerative diseases. Although antibiotics are mainly used to treat infections, new applications are being discovered. Reducing inflammation and mitochondrial dysfunction could break the vicious cycle triggered by these neurodegenerative diseases that ultimately lead to neuronal death. These mechanisms include mtUPR activation, iron chelation as well as the increase in autophagic flux and protease activity.

In this manuscript, we will review the experimental data supporting the potential benefits of several antibiotics for the treatment of numerous neurodegenerative conditions with mitochondrial involvement such as AD, PD, and HD (Table 1 and Figure 1).

## 2. Iron Accumulation and Lipid Peroxidation in Neurodegenerative Diseases

Iron accumulation and lipid peroxidation in different areas of the brain have been proposed as key disease-causing factors in many neurodegenerative diseases [119]. Abnormal iron homeostasis generally leads to iron overload, which destroys proteins and lipids via Fenton reactions [120,121]. Excessive iron accumulation and lipid peroxidation are frequently accompanied by oxidative stress, mitochondrial dysfunction, increased lipofuscin granules, and autophagy dysregulation [22,120,122]. Eventually, neuronal cell death occurs by ferroptosis, a cell death process dependent on iron-mediated lipid peroxidation.

Minocycline, a second-generation semi-synthetic tetracycline, is a known metal chelator [123], which is the reason why recent studies propose it as a potential treatment for brain iron overload [124]. In mice models, brain non-heme iron and brain iron handling protein levels decreased following minocycline treatment [56]. In fact, absorption of minocycline is significantly decreased by administration with iron supplements [125] and skin hyperpigmentation, a side effect of long-term minocycline therapy, may be related to insoluble minocycline–iron chelation products [126].

Minocycline can attenuate iron neurotoxicity in cortical neuronal cultures [127]. Chen-Roetling et al. treated cultured cortical neurons with ferrous sulfate causing significant neuronal death and increasing malondialdehyde levels, a canonical ROS and lipid peroxidation biomarker. However, minocycline treatment prevented this injury, with close-to-complete protection. Furthermore, the positive effect of minocycline was evaluated in iron-induced brain injury by intracerebral injection of iron in a rat model [56]. They found that minocycline attenuates iron-induced brain edema and BBB disruption. Recent studies showed that the combined treatment of deferoxamine and minocycline promotes a potent synergistic effect that reduces neuronal death, suppresses the activation of microglia/macrophages, and decreases iron accumulation, thus lessening brain damage, and improving neurological deficits [128]. These results suggest that minocycline’s metal-chelating properties are promising candidates for the treatment of diseases associated with iron overload.

Other tetracyclines, such as doxycycline, have also presented iron-chelating activity [123], but did not provide cytoprotection at effective minocycline concentrations. The fact that minocycline is considerably more lipid-soluble [129] than other tetracycline derivatives may enable its accumulation at higher concentrations in cell membranes that are vulnerable to iron-catalyzed lipid oxidation [25]. Moreover, in contrast to the iron chelator deferoxamine and doxycycline, minocycline increased cellular ferritin levels since the lipophilic minocycline-iron complex is sensed by the iron regulatory protein (IRP) that promotes ferritin expression [124,127]. Ferritin attenuates iron-mediated neuronal injury [128] by entrapping free iron molecules in its closed protein structure. Hence, induction of ferritin expression may also contribute to minocycline’s neuroprotective effect.

## 3. Neuroinflammation

Neuroinflammation is an inflammatory response within the central nervous system to events that interfere with tissue homeostasis and represents a common denominator in virtually all neurological diseases [130]. Activation of microglia, the main immune effector cells of the brain, contributes to neuronal injury by the release of neurotoxic products [130]. Toll-like receptor 4 (TLR4), expressed on the surface of microglia, plays an important role in mediating lipopolysaccharide (LPS)-induced microglia activation and inflammatory responses. Furthermore, several stress conditions can damage the outer and inner mitochondrial membranes and induce the release of mitochondrial components, such as mitochondrial mtDNA or cardiolipin [131]. These mitochondrial components are recognized as danger-associated molecular patterns (DAMPs), indicating cellular damage and thus eliciting innate immune responses [132]. Detection of both bacterial components, such as LPS, and mitochondrial DAMPs are pro-inflammatory signals. Notably, expression of most of these molecules is not restricted to specialized innate immune cells, such as macrophages, microglia, dendritic cells, or neutrophils, but also occurs in a large number of non-immune cells, including neurons. Antibiotic supplementation has been shown to regulate the neuroinflammatory response reducing its side effects [133,134,135,136].

Both clinical and laboratory studies have demonstrated the anti-inflammatory properties of minocycline [137,138,139,140]. Furthermore, their bioavailability is favorable since they are rapidly and completely absorbed, even in elderly populations, have a longer half-life, and present an excellent tissue penetration [141,142]. In addition, minocycline has a good safety record when used chronically, including dosages of up to 200 mg·day^−1^, the highest dosage recommended by the FDA [143]. Presymptomatic administration of minocycline inhibits neuroinflammation and glial activation in an ALS mouse model [48]. In fact, minocycline is a widely known strong inhibitor of microglial activation. Thus, minocycline is used in investigations on the polarization and pathogenesis of many diseases featuring microglial activation [144]. Generally, microglia polarization could be categorized into classical (M1) and alternative (M2) activation. The bacterial component LPS is known as a canonical M1 polarization inducer, and M1 microglia express proinflammatory molecules that include tumor necrosis factor-α (TNF-α), interleukin-1β (IL-1β), interferon-γ (IFN-γ), and nitric oxide (NO) as well as cell surface markers, CD86 and CD68. Although M1 microglia could be beneficial in the early stages of neurodegenerative diseases, their chronic activation could aggravate the pathological alterations and disease progression [145]. On the other hand, IL-4 induces microglia M2 polarization which can ameliorate chronic inflammation. M2 microglia express different molecules, such as IL-4, arignase1, Ym1, CD206, and IL-10, and show neuroprotective effects [146]. Kobayashi K et al. found that minocycline inhibited the expression of cell surface markers of M1-polarized microglia as well as the production of inflammatory cytokines (IL-1β, TNF-α, and IFN-γ) in vivo and in vitro. However, M2 marker expression was not affected. These results demonstrate the selective action of minocycline in microglia polarization.

Fluoroquinolones (FQs), one of the most important and commonly prescribed classes of synthetic antibiotics [64], have been shown to exert immunomodulatory activities by decreasing the production and release of inflammatory-associated cytokines, both in vitro and in vivo, in addition to their classical antimicrobial activity [65]. Zusso M et al. reported that FQs effectively reduce the release of IL-1β and TNF-α by LPS-stimulated primary microglia in vitro. This effect was achieved at a higher than clinically relevant concentration, but in agreement with some previous studies conducted in peripheral immune cells aimed at exploring the anti-inflammatory properties of these drugs [66,67]. Interestingly, the tested FQ concentrations did not produce any cytotoxic effect on microglia. This study shows that TLR4 is the primary target of the anti-inflammatory activity of FQs, which in turn decreased the inflammatory activity of LPS, resulting in the downregulation of LPS-induced inflammatory response via the TLR4/NF-κB pathway.

Rifampicin, a classic and safe anti-tuberculous drug, has neuroprotective effects in acute and chronic brain injury. Rifampicin may protect neurons via upregulating chaperone 78-kDa glucose-regulated protein (GRP78), enhancing sumoylation of α-synuclein, improving the UPR^mt^-related pathway PI3K/Akt/GSK-3β/CREB signaling or inhibiting neuroinflammation through TLR-4 pathway [80]. Furthermore, rifampicin can inhibit the release of IL-1β by suppressing the activation of NLRP3 inflammasome in microglia [79]. Liang Y et al. found that rifampicin could inhibit rotenone-induced microglia inflammation by promoting the clearance of damaged mitochondria through the autophagosomal/lysosomal pathway [147].

## 4. Alzheimer’s Disease

Alzheimer’s disease (AD) is a degenerative disease of the central nervous system with a high incidence in elderly people [148]. The main clinical manifestations are progressive memory loss, cognitive and language communication disorders, and personality changes [149]. Its main pathological features are the appearance of senile plaque (SP) and neurofibrillary tangles (NFTs) in patients’ brains. β-amyloid (Aβ) deposition and abnormally phosphorylated Tau protein deposition are the main components of SP and NFTs, respectively [150]. There is no effective treatment for AD presently available; however, Aβ is now a key established biomarker indicating the development of AD [151]. The aggregation Aβ in AD provokes mitochondrial dysfunction [152] and dynamics impairment, including mitophagy [92].

Aβ aggregates can infringe on mitochondrial damage [153]. When Aβ accesses mitochondria, it partly passes through the mitochondrial protein import apparatus through the translocase of the outer mitochondrial membrane 40 (TOMM40) protein pore [154]. The import is, however, not completed due to the presence of an Aβ acidic domain which clogs the import infrastructure and protrudes from the mitochondria into the cytoplasm causing severe mitochondrial dysfunction [155]. Damaged mitochondria release proinflammatory molecules such as mtDNA, adenosine triphosphate (ATP), cardiolipin, mitochondrial transcription factor A (TFAM), cytochrome c, formyl peptides, and RNA [156]. In relation to neuroinflammation, free mtDNA induces inflammatory signaling in astrocytes [157]. Mathew A. et al. demonstrated that adding mitochondrial oxidized polynucleotides to mouse primary astrocytes stimulates the expression of IL-6, monocyte chemotactic protein-1 (MCP-1), IL-1β, and TNFα [157]. mtDNA damage can be induced by hydrogen peroxide, and evidence indicates that this type of DNA oxidative damage is relatively specific to mitochondria [158]. The relationship between Aβ and mitochondria leads to the “mitochondrial cascade hypothesis” where Aβ, in any of its forms, does not contribute to AD dysfunction or degeneration *per se*. Rather, it induces aberrant mitochondrial function or cell bioenergetic states that trigger neurodegeneration, which in turn further promotes Aβ production [152]. Ruan et al. showed that mitochondria act as a sink for aggregation-prone proteins, which mitochondrial proteases degrade following their import [55]. A loss of this “mitochondria as guardian in cytosol” (MAGIC) function could in general promote protein aggregation [159].

The considerable efforts in the design of Aβ targeting molecules have been fruitful, providing several classes of compounds with different modes of action, but the drugs tested in clinical trials have given unsatisfactory results or caused adverse effects [160]. Salmona’s group proposes that tetracyclines promote Aβ degradation by proteases as well as inhibit aggregation and destabilize aggregates [161,162,163]. Interestingly, incubation of Aβ peptides with tetracycline led to the formation of colloidal particles that specifically sequestered oligomers, preventing the progression of the amyloid cascade. Therefore, they hypothesize that the internal structure of aggregates formed by Aβ peptides with tetracycline is non-homogeneous and governed by hydrophobic and charged multiparticle interactions. The formation of these supramolecular aggregates improves the solubility of Aβ peptides which correlates with the mechanism of action of small anti-amyloidogenic molecules [164].

Nevertheless, using doxycycline to treat AD patients in clinical trials is a highly controversial approach. One study proved that upon doxycycline treatment patients experienced a cognitive decline to a lesser extent [165] whereas no benefits were observed in another [166]. The two trials were comparable in terms of patients’ stages of disease at enrolment. Doxycycline was given orally at the dose of 200 mg/day together with rifampin 300 mg/day in the first study. The same doses were used in the second trial, with the sole difference that doxycycline was given at 100 mg twice a day, rather than once. The main differentiating factor between both studies was the duration of the treatment. In the former patients were treated for 3 months, whereas in the latter treatment continued for 12 months. As stated by the authors, one possible explanation for the outcome of the latter study is that doxycycline might have some detrimental properties that become apparent when the treatment is sustained for longer periods of time. In these studies, only the behavioral and functional aspects were examined, with no assessment of Aβ, tau levels, or inflammatory markers in the plasma and/or cerebrospinal fluid of the patients.

Minocycline was first tested in a drug-induced AD mice model by Hunter et al. [167]. They found that minocycline ameliorated cholinergic cell loss and reduced the simultaneous activation of microglia and astrocytes, leading to the transcriptional down-regulation of pro-inflammatory mediators and mitigating cognitive impairment. In amyloid precursor protein (APP) transgenic mice, minocycline suppressed microglial production of IL-1β, IL-6, TNF, and nerve growth factor but did not affect Aβ deposition [168]. In an AD rat model, minocycline treatment was able to correct behavioral impairments and lower levels of inflammatory markers and Aβ trimers in an early, pre-plaque inflammatory process [169]. Overall, minocycline treatment ameliorates the cognitive impairment and deficits in learning and memory that characterize AD in several models [143]; however, clinical trials with 200–400 mg/day of minocycline fail to delay the progress of cognitive or functional impairment in people with mild AD over a 2-year period [170].

## 5. Parkinson’s Disease

Parkinson’s disease (PD) is the second most common degenerative pathology of the central nervous system and its prevalence is higher among people over 65 years old, affecting 1–3% of the population [171,172]. The disease can present a wide range of manifestations. The most recognizable one is a mild tremor that might develop into unilateral resting tremors in the upper limbs over time. However, one out of four PD patients never develops tremors [171]. Other manifestations may involve somatomotor system dysfunction, as well as non-motor symptoms such as neuropsychiatric ones, sleep disorders, or loss of concentration [171,172]. Histopathologically, the presence of neuronal cytoplasmic inclusions called Lewis Bodies (LB), depleted dopamine levels, and loss of dopaminergic neurons in the substantia nigra pars compacta (SNpc) are characteristic features of this disease [171,172]. LB are mainly formed by the aggregation of phosphorylated α-synuclein in a misfolded fibrillary stage [90,171,173]. The formation of LB induces neuronal death which affects primarily the dopaminergic neurons in the SNpc [171,174]. The release of α-synuclein to the extracellular compartment might activate microglia and lead to the release of proinflammatory cytokines such as IL-1β through the activation of the NLRP3 inflammasome, thus amplifying neuronal damage [175,176]. Moreover, mitochondrial dysfunction plays a fundamental role in PD [171,172]. Ludtmann et al. suggested that monomeric α-synuclein has a regulatory function in mitochondrial bioenergetics by improving the efficiency of ATP synthase [90,177]. Furthermore, it has been described that aggregated α-synuclein disrupts the vesicular system and, consequently, mitophagy machinery, leading to the accumulation of dysfunctional mitochondria [173,178,179]. Mitochondrial dysfunction enhances ROS production, which may facilitate α-synuclein aggregation and would activate NLRP3 inflammasome [147,175,180]. Synaptic glutamate clearance is also compromised in PD: glutamate transporter-1 (GLT-1) is downregulated in patients causing an imbalance of glutamate homeostasis and excitotoxicity [102,181]. Currently, there is no curative treatment for PD. Most pharmacological formulations are designed to reduce the aggregation of α-synuclein, neuroinflammation, and mitochondrial dysfunction [90,182]. This has led to exploring the efficacy of existing molecules with potential activity against neuroinflammation, α-synuclein aggregation, or mitochondrial dysfunction for their application in PD. In this context, doxycycline has been identified as a potential treatment for PD, since it reduces neuroinflammation and oxidative stress, and prevents the aggregation of α-synuclein in animal models of PD [49,50].

Tetracycline antibiotics have already been used in non-infectious diseases such as acne vulgaris or rosacea at sub antimicrobial doses (20–40 mg/day) without serious side effects [175]. In fact, Egeberg et al. noticed a correlation between the use of tetracyclines in rosacea and a small reduction in the risk of developing PD [183]. Regarding microbiota, studies indicated there were no detectable effects on the normal oral bacteria or fecal or vaginal flora, the increase in the number of doxycycline-resistant bacteria, or the development of multi-antibiotic resistance following the treatment with sub antimicrobial dosage for 9 months [184,185].

Doxycycline treatment induces conformational changes of α-synuclein oligomers making them unable to form fibrils in vitro [49] and in cell-free models [173], but only in early elongated oligomers [49]. González-Lizarrafa et al. were able to establish a minimum dosage of treatment at 20–40 mg/day based on the aggregation kinetics of the antibiotic and α-synuclein in vitro, and the concentration of α-synuclein in cerebrospinal fluid (0.12 nM) [49]. More recently, Socias et al. found that the base of this anti-aggregation activity may be a structural motif in tetracycline that interferes with the aggregation of cross-beta structures [90]. On the other hand, Amaral et al. demonstrated that doxycycline activates the same signaling pathway as the nerve growth factor (NGF), hence promoting neuritogenesis. This effect may restore axonal and synaptic damage [50]. Minocycline has also been studied for its potential therapeutic use in PD. It plays a role in preventing apoptosis and reducing neuroinflammation by decreasing the mitochondrial inner membrane potential, thus diminishing the release of cytochrome c [173,175]. 

Rapamycin is another relevant molecule, which has offered positive results in animal models of neurodegeneration in terms of a decrease in neuronal loss and an improvement in the motor system by promoting autophagy via inhibiting mTOR [86,87,88]. This mechanism has been proposed for enhancing oligomer clearance and preventing α-synuclein aggregation [90,91]. In animal models, pretreatment with rapamycin has improved behavioral characteristics and inhibited the mTOR pathway [89].

Additionally, co-treatment of rapamycin and trehalose had shown an additive effect in promoting autophagy in vitro, which was later detected in vivo by Pupishev et al. by measuring changes in immunofluorescence of LC3-II in the substantia nigra. Interestingly, this effect was not observed in the striatum [87]. Unfortunately, this antibiotic presents significant long-term side effects such as lung toxicity, and an increased risk of type 2 diabetes, skin cancers, or lymphoma due to its immunosuppressive activity [186]. For this reason, further studies are required to establish a safer treatment either through the development of structural analogs [90] or the establishment of safer dosages [186]. In this respect, Gonzalez-Alcocer et al. validated a non-toxic dosage in murine PD. Said dosage proved to be neuroprotective against dopaminergic neuron loss and, after 14 weeks of treatment, no evidence of significant tissue damage was observed in different solid organs [186].

Rifampicin has also been studied as a potential treatment for PD. As mentioned before, rifampicin effectively reduces α-synuclein aggregation and exerts anti-neuroinflammatory activity [147]. It is also thought to play a role in the suppression of the NLRP3 inflammasome activity, leading to a decrease in IL-1b release. This occurs due to the suppression of proinflammatory pathways such as NF-κB, phosphorylated MAPKs, and TLR-4s [79,147,187]. Suppression of the NLRP3 inflammasome activity by rifampicin may have a role in the inhibition of apoptosis in neurons since it seems to upregulate chaperone GRP78 and reduce the aggregation of α-synuclein [90,91]. Rifampicin anti-inflammatory activity was analyzed in a study with several PD cell models, where pre-treatment with rifampicin induced autophagy. Enhanced autophagy led to an increase in the number of lysosomes and decreased mitochondrial injury and ROS production. Thus, rifampicin preserved mitochondrial function and reduced the expression of pro-inflammatory cytokines, IL-1 and IL-6, and microglial inflammation [147]. However, the addition of chloroquine, an autophagy inhibitor, abolished the reduction of pro-inflammatory cytokines. A later study confirms that cell viability was compromised upon chloroquine addition [81,147]. Furthermore, Yurtsever et al. further proved that pretreatment with rifampicin improved the maturation of autolysosomes and reduced autophagosomes in a PD cell model [81]. Interestingly, this was related to a reduction in microglia-related inflammation. Moreover, the autophagy-promoting activity of rifampicin is dose-dependent [147] and does not compromise cell viability at the highest concentration of 100 μM. In animal models, rifampicin improved the motor system activity in a zebrafish PD model [180].

Ceftriaxone is a third-generation cephalosporin, with neuroprotective features and limited side effects [90]. It has been reported that ceftriaxone binds to the C-terminal region of monomeric α-synuclein and inhibits its aggregation and fibril formation [90,100,101]. Ceftriaxone also upregulates GLT-1 expression in substantia nigra astrocytes, leading to increased glutamate uptake. This uptake has been linked to reduced loss of dopaminergic neurons by decreasing hyperactivity and excitotoxicity [102,103,104,105]. Ceftriaxone also decreased the activation of glial cells through the downregulation of proinflammatory TLR4 and NF-κB pathways in PD models [104]. Subsequent studies in PD mice models confirmed that a 5 mg/kg/day ceftriaxone reversed neuronal loss in the substantia nigra and the hippocampus by enhancing neurogenesis [106,107], which consequently ameliorated neuronal deficits [107,181]. Ceftriaxone prevented the decrease in cell viability after 1-methyl-4-phenylpyridinium (MPP+) exposure in vitro [105,188]. Moreover, ceftriaxone reduced the adverse effects of levodopa dyskinesia, the standard treatment for PD, with no impact on levodopa effects [102]. Huang et al. also found a synergistic neuroprotective action of ceftriaxone and erythropoietin in PD mice models [189].

The search for alternative targets to treat PD has led to the study of other antibiotics such as geldanamycin, an Hsp90 inhibitor that boosts the activity of Hsp70 and Hsp40 which are involved in the solubilization of protein aggregates. Geldanamycin treatment has been reported to prevent α-synuclein aggregation in the early stages of disease progression [90,114]. Nonetheless, its significant cell toxicity has led to the development of safer molecules such as 19-Ph-GAt [190]. Another promising therapeutic target is PTEN-induced kinase 1 (PINK1), since its mutation is known to cause the early onset of PD [191,192]. Niclosamide, a salicylanilide drug used to treat parasitic infections, promotes PINK1 activation by driving mitochondrial depolarization [191,192]. Alternatively, other approaches involve chloramphenicol treatment. This drug binds the 50S subunit of the ribosome and inhibits protein translation, which in turn causes a decrease in mitochondrial metabolism and ROS production [182]. Chloramphenicol treatment had a protective effect when SN4741 dopaminergic neuronal cells and rat primary cultured dopaminergic neurons were exposed to paraquat [182].

Whilst it is a promising field of study, most of the molecules proposed above were mainly effective as preconditioning treatments in PD biological models. Hence, their application to patients with advanced PD might have a poor outcome in clinical trials. Moreover, some of these drugs presented side effects, which is the reason why further studies exploring safer derivatives molecules are needed [193].

## 6. Huntington’s Disease

Huntington’s disease (HD) is an autosomal dominant inherited neurodegenerative disease characterized by progressive motor, behavioral, and cognitive decline [194]. The disease results from a CAG trinucleotide repeat expansion in the huntingtin gene (HTT) on chromosome 4. Although its driver gene was discovered in 1993, the pathophysiology of this disease remains mostly unknown [195]. The huntingtin protein is widely expressed and has many functions in human neurons [196] such as intracellular protein trafficking regulation [197], protein scaffolding [198], and synaptic vesicle formation [196,199].

Overall, there is an average of 17–20 CAG repeats in the HTT gene but with 40 or more CAG repeats, HD emerges with full penetrance. There is still no therapy to slow the neurodegeneration or the progressive functional loss rate [52,53,142,162,200,201,202,203,204]. In HD, there is a prominent imbalance between mitochondrial fission/fusion events. Mitochondrial fission is prevalent, as evidenced by the increase in Drp1 and Fis1 protein levels as the disease progresses. On the contrary, mitochondrial fusion proteins such as Mfn1/2 and Opa1 displayed low expression levels [205]. In fact, in vivo and in vitro studies showed that binding of the mutant HTT to Drp1 acts as the main trigger of the fission process in HD models [206]. Alternatively, mutant HTT has the ability to enhance Drp1 activity by posttranscriptional modification [207]. Usually, cells disrupt the mitochondrial network to remove defective mitochondria by mitophagy and thus protect themselves against apoptosis-initiating agents. However, the disruption promoted by mutant HTT in mitochondrial dynamics can lead to neuronal death and apoptosis [208]. Several studies have tried to either boost mitophagy or overexpress Mfn2 with the aim to modulate the mitochondrial dynamics process and reduce apoptosis in neurodegenerative diseases [209,210]. Nonetheless, some authors [211] claim that ATP depletion in HD is due to low mitochondrial biogenesis rather than increased mitochondrial disruption. In fact, HD patients present decreased transcripts and protein levels of PGC1α [212] which is essential for mitochondrial biogenesis [213]. The relationship between PGC1α and HD lies in the altered CREB/TAF4-dependent transcriptional pathway critical for the regulation of PGC-1α gene expression [212]. Thus, mutant huntingtin represses PGC-1alpha gene transcription. In turn, decreased mitochondrial biogenesis translates into enhanced anaerobic metabolism in affected cerebral areas of HD patients, where excessive lactate generation ultimately leads to cell death [214].

Paldino E. et al. showed that the administration of doxycycline was protective in the R6/2 HD mouse model in terms of survival, motor performance, and neuroprotection. Interestingly, these effects correlated with a significant decrease in microglial activation [142]. In addition to reducing microglial activation and exerting an anti-inflammatory function, doxycycline promoted CREBs activity and the expression of brain-derived neurotrophic factor (BDNF). It has been postulated that HD neuronal impairment could be caused by a transcriptional dysregulation of the cAMP and CREB signaling cascades [215,216]. Therefore, preventing the decrease in cAMP signaling and the loss of CREB-regulated gene transcription represents a valid therapeutic strategy for HD [217]. CREB induces transcription of thousands of target genes, some of which are related to the UPR^mt^ [218,219]. The role of the CREB protein family in the central nervous system has been shown to support neuronal survival [220], regulate neuronal migration [221], modulate synaptogenesis [222], and contribute to the formation of long-term potentiation and memory [223]. One of the key downstream mediators in this context is BDNF, whose expression is highly altered in HD [224]. Despite being a promising therapeutic agent, no clinical trials examining the efficacy and safety of doxycycline as a treatment for HD have been carried out.

Minocycline also blocks mTOR activity which is connected to NF-κB [225] and eNOS/iNOS activity [226]. mTOR inhibition may contribute to the anti-inflammatory and cytoprotective effects of this second-generation tetracycline. Moreover, minocycline blocks the expression of caspase 1 and 3 in an HD mouse model [54]. Given these promising features, Thomas M. et al. started a clinical assay with minocycline treatment for HD patients [227]. They demonstrated that minocycline is a safe and tolerable medication for HD patients, without significant adverse events or adverse drug interactions. However, they did not find any neurological improvement in the patients, which they attributed to the short duration of the clinical trial, which only lasted for 6 months. Contrary to their belief, a more recent clinical trial also showed no effect on the progression of the disease [228], suggesting that tetracycline treatments may require a synergistic effect with another molecule to be effective. An example of this tetracycline synergistic effect is found with creatine in an ALS mouse model [229].

## 7. Amyotrophic Lateral Sclerosis

Amyotrophic lateral sclerosis (ALS) is a progressive neurodegenerative disease that primarily affects the neurons responsible for controlling voluntary muscle movement [230]. ALS affects approximately 4–8 out of 100.000 individuals, with a prognosis for survival of 2 to 5 years. Abundant abnormal protein aggregations have been found in the neurons of CNS in both ALS patients and animal models of ALS [231]. The ubiquitin-proteasome system [232] and the autophagosome-lysosome pathway, including mitophagy, are the most important degradation machinery to clear the aggregated proteins [233]. As seen previously, the balance between protein aggregation and degradation is involved in the pathogenesis of several neurodegenerative diseases [234]. Most ALS-associated identified genes have been functionally implicated in autophagy and/or mitophagy, specifically in the clearance of protein aggregates and/or damaged mitochondria [235]. ALS genes known to function directly in autophagy include OPTN, TBK1, and SQSTM1. Moreover, proteins encoded by the genes C9ORF72, VCP, CHMP2B, VAPB, ALS2, SOD1, and DCTN1 have all been related to vesicular trafficking and may affect autophagy either directly or indirectly [236,237].

Accumulation of damaged or dysfunctional mitochondria is a contributing factor in ALS. Moore AS and Holzbaur ELF showed OPTN translocation to mitochondria after mitochondrial uncoupling [238]. However, OPNT translocation to damaged mitochondria was defective in a mutant OPTN cell model [238]. Similar findings were observed in an ALS-linked mutant TBK1 model [238]. Therefore, loss-of-function OPTN or TBK1 mutations result in impaired mitophagy and accumulation of damaged mitochondria. Thus, inefficient clearance of damaged mitochondria in ALS-associated mutants could be a contributing factor leading to mitochondrial dysfunction and accumulation, a prevalent feature in the motor neurons of ALS patients. In fact, the ablation of genes involved in autophagy is enough to trigger neurodegeneration in murine models, including multiple ALS-related genes which are directly involved in mitophagy such as OPTN, TBK1, or SQSTM1 [239].

Considering these findings, one potential therapeutic approach for ALS would be to enhance autophagy/mitophagy activity by rapamycin supplementation. However, Zhang X et al. demonstrated that rapamycin accelerated disease progression and neuropathological processes in the mutant SOD1 ALS mice model through the activation of the apoptotic machinery [240]. Although they observed increased autophagy initiation, neurons were unable to clear the accumulation of autophagosomes. On the other hand, Castillo K et al. showed that trehalose, a chemical chaperone that specifically engages mTOR-independent autophagy, increased life span and attenuated disease progression in an ALS mice model [241]. They concluded that stimulation of mTOR-independent autophagy represents an interesting approach for the future development of therapeutic strategies to treat ALS.

The vast number of mutations that cause ALS makes the pathology extremely difficult to treat, due to the obvious incongruences between treatments depending on the mutation. Finally, a study performed in Sweden conclude that the chronic use of antibiotics is associated with an increased risk of ALS [242].

## 8. Prion Diseases

Prion diseases are fatal neurodegenerative disorders with a highly spreading nature. The infectious agent causing prion disease, known as PrP^Sc^, is a pathogenic misfolded and aggregated form of the cellular prion protein, PrPC [243]. Following transmission to a naive host, prions seed the misfolded form of host PrPC in an autocatalytic process, leading to an exponential increase in PrP^Sc^ in the brain and the spinal cord that eventually leads to neuronal death [244]. Prions are highly stable and accumulate in the central nervous system from months to years, eventually triggering neurodegeneration and neuronal loss as well as astrocytes and microglia activation [245]. The incubation period of the disease is exceptionally variable and may last from years to weeks. Common symptoms include behavior abnormalities, motor dysfunction, cognitive impairment, and ataxia, depending on the prion type [246]. No therapy is currently available beyond palliative care.

Mitochondria of prion-infected animals show morphological and functional abnormalities in the CNS [247]. Alterations in calcium homeostasis related to mitochondria and endoplasmic reticulum dysfunction are typical in prion diseases [248]. Increased calcium concentration promotes mitochondrial membrane loss, enhanced ROS generation, and reduced ATP production, ultimately leading to cellular apoptosis [249]. Taken together these findings suggest that mitochondrial dysfunction may contribute to the neurodegeneration observed in prion diseases. Choi et al. showed that mitochondrial fusion was upregulated in whole brains from prion-infected mice, and that expression levels of mitochondrial fission protein 1 (Fis1) and mitofusin 2 (Mfn2) were elevated in the hippocampus and striatum [250]. Furthermore, the expression of the mitochondrial fission-related protein, Drp1, was significantly reduced in the hippocampus. By inhibiting mitochondrial fission, the mitochondrial network cannot be repaired, which led to a decrease in mitochondrial mass in neurons, most of which were degenerated. These abnormalities were detected in at least four different prion disease mouse models [251]. Despite cellular Drp1 protein levels being decreased in prion-infected neuronal cells both in vitro and in vivo, the levels of the mitochondrial fission protein DLP1 were increased in some prion models. This imbalance results in extensive mitochondrial fragmentation and dysfunction, as well as neuronal death and decreased synaptic plasticity [252].

Nowadays, several classes of antibiotics such as tetracyclines or polyenes are known to prevent aggregation of PrP^Sc^ and delay the onset of prion diseases. Syrian hamsters, a prion disease model, when treated with tetracycline showed a delay in the appearance of symptoms due to inactivation and decreased accumulation of prion protein [253]. Hannoui S et al. reported a reduced accumulation of PrP^Sc^ in neurons derived from infected mice after tetracycline, doxycycline, and minocycline treatment [254]. In infected mice, doxycycline treatment also decreased the levels of PrP^Sc^ to the extent that neuronal dysfunction was no longer observed [51]. Some clinical trials noted that doxycycline therapy might increase the life expectancy of patients [255,256]. Alternatively, Amphotericin B decreases the accumulation of PrP^Sc^ in neuroblastoma cells by manipulating the lysosomal trafficking of PrPC and preventing its intracellular conversion [117]. MS-8209 (N-methyl glucosamine salt of 1-deoxy-1-amino-4,6-benzylidene-D-fructosyl-AmB) suppresses the accumulation of PrP^Sc^ in mice spleen [257]. Poylene antibiotics show a strain-specific effect on the aggregation of PrP^Sc^ and interact directly with neurons, increasing the life span of infected animals [258]. Rapamycin, which theoretically improves mitochondrial dynamics, shows an increase in autophagy and clearance of PrPC in mice models of Gerstmann–Straussler–Scheinker disease and prevents plaque formation, but is unable to prevent neuronal damage [259].

## 9. Primary Mitochondrial Diseases

Mitochondrial diseases are a set of highly heterogeneous disorders caused by mutations in either nuclear or mitochondrial genes that primarily affect oxidative phosphorylation and ATP synthesis. These conditions are the most common group of inherited metabolic diseases and one of the most common types of neurological disorders [260,261,262]. In fact, most mitochondrial disease patients present prominent neurologic and myopathic disorders [263]. It is widely known that neurons have a high energy demand and critically depend on mitochondria to maintain synaptic transmission through the regulation of ATP and calcium levels [264]. Early onset mitochondrial diseases are severe clinical entities often caused by autosomal recessive mutations in nuclear genes (nDNA). Examples of these include Leigh syndrome, caused by mutations in the mitochondrial oxidative phosphorylation (OXPHOS) complexes and their assembly factors [265], and Alpers–Huttenlocher syndrome, due to nDNA mutations in the mtDNA polymerase gene [266]. Other high-energy demanding tissues such as the skeletal and cardiac muscles can also be affected in patients with these diseases.

Mitochondrial diseases diagnosed in adulthood affect around 1 in 4300 adults, and are mostly caused by mtDNA mutations (approx. 87% of cases) [267]. Mitochondrial syndromes caused by mtDNA mutations that affect the nervous system include Leber’s hereditary optic neuropathy (LHON), myoclonic epilepsy with red-ragged fibers (MERRF), and mitochondrial encephalopathy, lactic acidosis, and stroke-like episodes (MELAS) [268].

Given that antibiotics are known to impair mitochondrial function in mammalian cells, several studies have focused on assessing the impact of antibiotic treatments on mitochondrial function in neurons. According to Xiao et al. metronidazole, tigecycline, azithromycin, and clindamycin, but not ampicillin or sulfamethoxazole, induced apoptosis in both primary neurons and neuronal cell lines at clinically relevant concentrations. Moreover, they demonstrated that tigecycline, azithromycin, and clindamycin trigger cell death through oxidative damage whereas metronidazole does so in a ROS-independent manner [269]. These results go in line with previous evidence that bactericidal but not bacteriostatic antibiotics induce oxidative stress and damage in mammalian cells [39]. Additionally, tigecycline, azithromycin, and clindamycin were reported to cause mitochondrial dysfunction in neurons. Interestingly, this effect was abrogated when they were co-administered with an antioxidant.

At first sight, the use of antibiotics to treat mitochondrial diseases seems paradoxical and controversial because, as previously exposed, they may interfere with mitochondrial function. Nonetheless, Perry E.A. et al. showed that the tetracycline antibiotics family increased cell survival and fitness in MELAS cybrids and Rieske cells (Knockout Complex III mouse fibroblasts) under glucose restriction [270]. Specifically, doxycycline improved survival in wild-type cells treated with piericidin (complex I inhibitor) or antimycin (complex III inhibitor) during glucose deprivation. In addition to tetracyclines, the anti-parasitic agent pentamidine and the antibiotic retapamulin also scored positive on the screening in MELAS and mutant ND1 cybrid cells. To support their findings, the authors propose that antibiotics exert a “mitohormetic effect” via UPR^mt^ induction. Mitohormesis is defined as the mechanism through which mild mitochondrial stressors activate cytoprotective mechanisms resulting in enhanced mitochondrial stress resistance [271,272]. Given the bacterial origin of mammalian cells’ mitochondria and the fact that they conserve prokaryotic features such as the 55S or 60S ribosomes, it is no wonder that mitochondria are exceptionally sensitive to antibiotics [40]. By partially inhibiting mitochondrial translation antibiotics can activate mitohormesis, thus triggering a retrograde signaling response including the modulation of mitochondrial dynamics, the expression of nuclear and mitochondrial-encoded genes, and an antioxidant response, stimulating mitochondrial function and boosting cellular defense mechanisms that increase stress resistance [273]. Mitohormesis has been closely linked to UPR^mt^ activation [274], metabolite and ion disbalance [275], and ROS production [276].

Suarez-Rivero et al. also demonstrated that tetracycline treatment boosts the production of UPR^mt^-related proteins and promotes the activation of pathways involving cAMP and cGMP [277], which might be involved in mitochondrial compensatory mechanisms comprising sirtuins and chaperones’ activity [278,279,280]. Tetracycline treatment and the subsequent activation of UPR^mt^ lead to an increased number of chaperones and mitochondrial auxiliary proteins that promoted the stability of mutant mitochondrial proteins which would carry out their function to some extent [277].

The activation of mitochondrial compensatory mechanisms has been associated with the regulation of mitophagy and mitochondrial biogenesis as well as with a beneficial mild induction of ROS signaling pathways. Animals presenting impaired cardiac mitophagy and a consequent accumulation of damaged ROS-overproducing mitochondria develop cardiomyopathy. It has been observed that this condition can be improved through ROS-dependent activation of mitophagy, which can act as a mitochondrial quality control mechanism to prevent vicious cycles of ROS formation and mitochondrial dysfunction [281]. Furthermore, muscle-specific knockout mice for COX15, a complex IV assembly protein, are able to express alternative oxidases (AOX) that bypass respiratory complexes III and IV, thus transferring electrons directly to oxygen. In doing so, they exhibit decreased ROS generation, PGC-1α signaling activation, and increased lifespan [282]. Livers from adult mice depleted from superoxide dismutase 2 during embryonic development display mitochondrial adaptive responses with increased mitochondrial biogenesis and antioxidant defenses and decreased ROS levels [283]. Taken together, these results show the impressive ability of mitochondria to adapt to cellular insults.

There is no proven treatment for most primary mitochondrial diseases, only palliative therapies. Triggering mitohormesis with antibiotics such as tetracyclines or their derivatives could open the door to new therapeutic perspectives for these severe pathologies.

## 10. Cerebral Ischemia

Cerebral ischemia (CI) and its implications are currently one of the leading causes of mortality and morbidity worldwide. Given that the brain is one of the most energy-consuming organs, disruption of the blood supply, and thus of nutrient and oxygen availability, can result in severe neuronal damage [284]. There is increasing evidence that acute neuronal damage induced by cerebral ischemia promotes protein aggregation, suggesting a connection between the pathomechanisms of neurodegenerative diseases and stroke [285,286,287]. Thus, it seems that brain ischemia contributes to the development of Alzheimer’s disease-like neurodegeneration through various mechanisms, including accumulation of amyloid protein precursor, tau protein phosphorylation, neuroinflammation, dysregulation of Alzheimer-related genes, neuronal cell death, synaptic dysfunction, white matter alterations, and brain atrophy [288,289].

Apart from preventing the risk factors associated with ischemic events, therapeutic resources are limited. The only available treatments for ischemia up to the present are thrombolytic drugs such as tissue plasminogen activator (tPA). The efficacy of this therapy is limited by the reduced time window at which they can be administered, in the hours following the ischemic episode [290]. The development of new therapies for the treatment of CI is therefore urgent. Deprivation of blood flow triggers pathophysiological mechanisms that disrupt cellular homeostasis, including excitotoxicity, oxidative stress, inflammation, apoptosis, and cell death. In the context of CI, mitochondrial dysfunction has been identified as one of the key cornerstones, due to its involvement in the pathological pathways associated with ischemic stroke. Thereby, mitochondria are emerging as a therapeutic target in the search for potential novel therapies in CI [291]. However, as the development of new drugs for CI is complex, the repositioning of existing drugs such as antibiotics appears an attractive approach in terms of economic investment and timelines [292]. Thus, Lu et al. confirmed that a two-week treatment with minocycline could prolong survival and promote functional outcomes after CI by alleviating neuronal injury and reactive gliosis [293]. Furthermore, they were able to show that minocycline promotes M2 polarization of microglia and inhibits M1 polarization, by the shift of STAT1 to STAT6 pathway, leading to neuronal survival and neurological functional recovery in vitro and in vivo [293]. Camargos et al. assayed a CI-induced mice model. They verified that intraperitoneal injection of 30 mg/kg minocycline for 14 days resulted in antidepressant and anxiolytic effects in comparison with the control group, which experimented with depressive and anxiety-like conduct. In addition, they demonstrated that the minocycline treatment ameliorated hippocampal lesions, resulting in a reduction of infarct area and ischemic neurons in mice [294]. Yamasaki et al. used PET to assess the sole and combined treatment outcomes of minocycline and KML29, a strong monoacylglycerol lipase inhibitor (MAGL-i), in ischemic-injury rat models. Intravenous administration at doses of 10 mg/kg and 1 mg/kg, respectively, over 4 days were assayed. The aim was to exploit the neuroprotective benefits of both drugs in CI. Interestingly, they showed different neuroprotective properties. Minocycline targeted oxidative stress induced-neuroinflammation, observed in hypoxic regions. Meanwhile, KML29 showcased neuroprotective effects in the striatum, targeting glutamate neuroexcitotoxicity by inhibiting neuronal apoptosis [295].

In addition, glutamate-mediated excitotoxicity, an important mechanism leading to postischemic stroke injury, was ameliorated by ceftriaxone, a beta-lactam antibiotic, which is able to stimulate the expression of the major glutamate transporter GLT1. As glutamate release comprises one of the pathological mechanisms of ischemia, this action is notably valuable [292]. Thus, Smaga et al. reported that a dose of 200 mg/kg of ceftriaxone for a minimum of 2 days restored GLT1 expression [296]. Likewise, Krzyzanowska et al. demonstrated that pre-treatment with ceftriaxone and N-acetylcysteine, 200 mg/kg and 150 mg/kg respectively for five consecutive days, modulates GLT-1 expression and improves survival in a model of focal cerebral ischemia in rats [297].

## 11. Neuropsychiatric Diseases: Schizophrenia and Depression

This section is included in this review since major psychiatric and neurodegenerative diseases share genetic susceptibility and pathophysiology [298]. Thus, many causal proteins and interacting proteins, and the central role of synaptic transmission, immune and mitochondrial function are processes that participate in the shared pathogenesis.

Schizophrenia is a chronic neuropsychiatric disease that affects around 1% of the world population [299]. It may present with a wide range of symptoms either classified as positive (delusion, hallucination, and paranoia) or negative (amotivation, anhedonia, avolition, asociality, and flat affect). Negative and positive symptoms are caused by different alterations in neurotransmitter functions. Positive symptoms are caused by hyperfunction in the mesolimbic dopamine pathway [300], whereas negative symptoms may include abnormal GABAα receptors, disruption of glutamatergic systems, and abnormal microglial activation [299]. Pathophysiologically, neuroinflammation plays a key role in the disease, as evidenced by the high microglial activity that has been found in these patients [301,302]. This altered function of the brain immune system has been related to the dysfunction of glutamatergic and dopaminergic neurotransmissions [303], as well as to gray matter volume loss [302]. On the other hand, energy metabolism is also altered in the brain of patients with schizophrenia. This alteration may be due to mitochondrial dysfunction increased lactate levels and acidemia found in patients’ brains [304]. This alteration may also affect neurotransmission, since dopamine levels and NMDA receptor activity are highly sensitive to pH changes [304]. Furthermore, an elevated synthesis and release of dopamine in presynaptic neurons has been found, suggesting that presynaptic mitochondria are exposed to higher levels of dopamine, a complex I inhibitor. Thus, it generates a decrease in ATP synthesis without increasing oxidative stress [300,304]. Furthermore, there are alterations in the expression of genes related to energy metabolism in the brain of schizophrenia patients [304]. Although there is no cure for schizophrenia, it is usually treated with dopamine receptor antagonists [300] which control positive symptoms but have little to no effect on negative symptoms [304,305,306].

Different studies link high levels of pro-inflammatory cytokines to an increased risk of depression or psychotic disorder [307,308]. Thus, adolescents with higher serum levels of pro-inflammatory cytokines have an increased risk of developing schizophrenia [308]. This may be due to intense synaptic pruning by microglia during this age that contributes to the observed reduction in synapse density in this disease [309].

The most studied antibiotic molecule for the treatment of schizophrenia is minocycline, whose neuroprotective properties have been previously described in this review. Not only does it inhibit microglia activation, but also pro-apoptotic caspases or cytochrome c release [301]. Xiang et al. found in a meta-analysis that minocycline treatment improved positive symptoms in schizophrenia [310]. However, no improvements were observed in attention focus, working memory, visual and verbal learning, or problem-solving [310]. Several studies have found high-dose minocycline treatments to improve negative and cognitive symptoms and reported a correlation to decreased IL-1β and IL-6 serum levels. These observations support the role of neuroinflammation in the development of cognitive impairment and negative symptoms in the disease [299,301,311,312]. The addition of minocycline to regular antipsychotic treatment such as risperidone and clozapine also improved psychotic symptoms, working memory, avolition, and anxiety/depression [301,303,310,313]. Furthermore, Wehring et al. observed decreased pro-inflammatory cytokine levels in patients treated with both risperidone and minocycline [299]. Interestingly, a recent study on the synergistic effect of minocycline and clozapine reported abnormally high plasma clozapine levels following minocycline addition, although the explanation for this phenomenon is unclear [313]. Minocycline’s ability to lower microglial engulfment of complement opsonized synapses was confirmed by Sellgren et al. [309]. They also confirmed the link between minocycline or doxycycline treatment in adolescents with a lower risk of developing posterior psychotic episodes [309].

However, the benefits of minocycline treatment in schizophrenia are controversial. There are marked discrepancies between studies reporting no effects after completion of the treatment and others supporting an improvement of cognition and positive symptoms [314,315]. These divergencies may be due to different trial designs, the stage of the disease at which patients are treated, the type or the severity of the symptoms, treatment length and dosage, the duration of follow-up, or the sample size. Moreover, schizophrenia does not always respond in the same way to antipsychotic treatments and so could happen with minocycline. It has been proposed that minocycline may only be effective at the initial stages of the disease [310,314]. On the other hand, it should be noted that minocycline could have associated toxicities, since it has been related to the development and worsening of autoimmune diseases [312].

Doxycycline was found to have a similar effect to minocycline in improving cognitive and positive symptoms in mice models of schizophrenia. It was reported to prevent and reverse decreased social interaction, confirming the antipsychotic-like activity. Ketamine-induced models treated with doxycycline had higher levels of reduced glutathione, catalase, and superoxide dismutase. Furthermore, treatment with doxycycline-enhanced risperidone (an inhibitor of dopaminergic and serotonergic pathway activity in the brain) effects by lowering locomotion activity, which suggests interference with dopaminergic transmission in mice models [316].

Patients with schizophrenia often present signs of depression. Some studies suggest a shared mechanism between the two diseases [302,317]. Neuroinflammation, oxidative stress, and dopaminergic imbalance are known to play a role in depression [318]. Furthermore, higher counts of myeloid and CD4+ T cells were found in a lipopolysaccharide model of depression [318]. Interestingly, minocycline treatment decreased proinflammatory cytokines and reduced swim latency in the Porsolt swim test, suggesting both inhibition of microglia activation and amelioration of depressive symptoms [319,320].

Minocycline was also found to ameliorate depressive symptoms in a clinical trial with bipolar depression patients in an 8-week treatment study, albeit no significant cognitive improvement was identified. It was also found to trigger a reduction in IL-12p70 levels, a cytokine for Th1 cell differentiation related to reduced inflammation in bipolar disorder [321]. Doxycycline has been proven to improve depressive behavior in mice models by increasing reduced glutathione [318]. The combined use of doxycycline and escitalopram improved its anxiolytic effect [318]. However, anhedonic behavior did not improve with the treatment.

Tetracyclines’ application to treat neuropsychiatric disorders may be of interest given their ability to attenuate immune responses and block damage due to mitochondrial dysfunction and ROS [318]. Nevertheless, neuropsychiatric diseases are not sufficiently studied. The lack of a wide variety of drugs may well be due to insufficient knowledge about the pathophysiological mechanisms of schizophrenia. Further research should be conducted in the field to test new drug alternatives.

## 12. Conclusions

**Figure 2:**
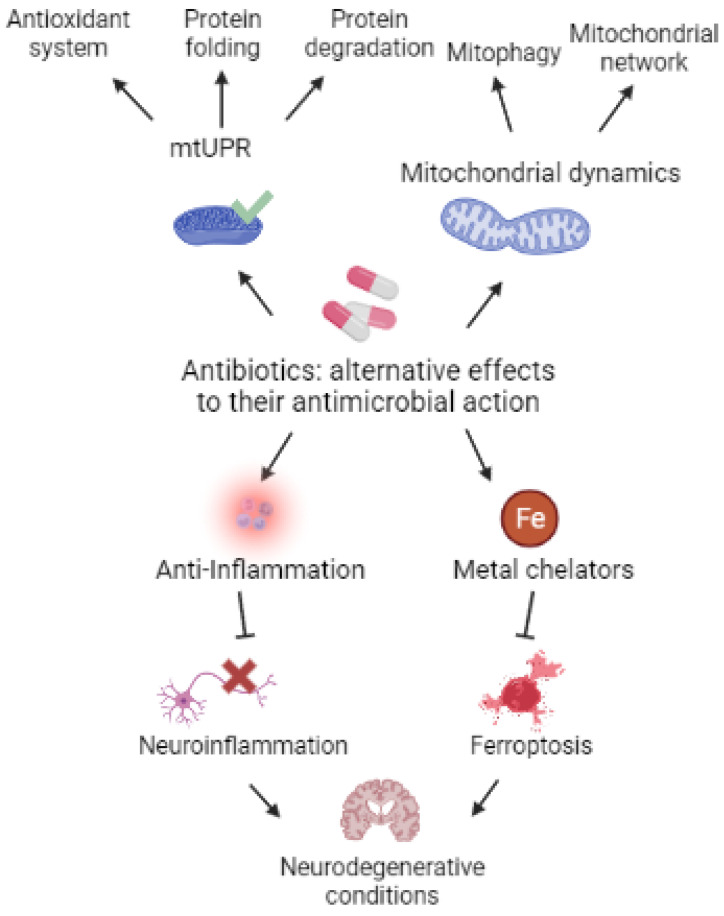
Alternative effects of antibiotics apart from their anti-microbial activity.

As presented in this review, the etiology of neurodegenerative diseases is extremely complex and can be etiologically diverse. However, most of these diseases share the common feature of mitochondrial dysfunction. The application of antibiotics to treat mitochondrial insults has always been controversial for plenty and reasonable reasons [38]. However, not all antibiotics function in the same way or at the same dosage. Thanks to their pleiotropic effects, these drugs may open new possibilities for the treatment of neurodegenerative disease beyond their antimicrobial activity (Figure 2). However, more research is needed to address the potential side effects of their chronic administration such as the risk of dissemination of antibiotic-resistant pathogenic strains. For this reason, the development of antibiotics derivatives without antimicrobial activity but retaining their neuroprotective properties will be another interesting research field in the future.

## Figures and Tables

**Table 1:** Antibiotics and their alternative therapeutic targets.

Antibiotic	Standard Application	Potential Effect	Clinical Trials	Side Effects
Tetracyclines family [47]	Broad spectrum bacteriostatic Bacterial ribosome inhibitor	Anti-inflammatory (Microglial M1 inhibition) [48] α-synuclein complex [49,50], prion [51,52], and β-amyloid peptide [53] aggregation inhibition Neuritogenesis promoter [50] UPR^mt^ activator [54] Increases mitochondrial protease activity [55] Iron chelator [56]	Huntington’s disease [57] Aneurysms and cerebral arteriovenous malformations [58] Pancreatic cancer [59] Degradation and permeability of collagen membrane [60] Alzheimer’s disease [61,62]	Gastrointestinal and skin adverse effects, drug-induced lupus, hypersensitive syndrome reaction [63]
Fluoroquinolones family [64]	Broad spectrum bactericidal Multidrug resistant bacteria	Anti-inflammatory (TLR4/NF-κB pathway) [65,66,67]	Bladder cancer [68] Crohn’s disease [69] Chronic obstructive pulmonary disease [70]	Tendinopathy [71], aortic diseases [72], gastrointestinal effects [73], psychiatric adverse reactions [74], seizures, confusion/encephalopathy [75,76], dysglycemia [77]
Rifampicin [78]	Gram positive bactericidal Tuberculosis, leprosy, and legionnaire’s disease treatment	Anti-inflammatory (TLR4 and NLRP3 pathway) [79,80] UPR^mt^ activator [80] Chaperone enhancer [80] α-synuclein sumoylation [80] Improves autophagy flux [81]	Alzheimer’s disease [61] Diabetes [82] Metabolism homeostasis [83]	Cutaneous reactions, gastrointestinal effects, hepatitis, and thrombocytopenia [84]
Rapamycin [85]	Natural anti-fungal antibiotic used as an immunosuppressor	mTOR inhibitor (Autophagy enhancer) [86,87,88,89] Reduce α-synuclein aggregation [90,91] Mitochondrial clearance [92]	Alzheimer’s disease [93] ALS [94] Aging [95] Myelodysplastic syndrome [96] Metabolism homeostasis [97]	Anemia, hyperglycemia, dyslipidemia, renal, pulmonary, and dermatologic adverse effects, angioedema, osteonecrosis, and lymphedema [98]
Ceftriaxone [99]	Post-surgery infections Multidrug resistant bacteria	α-synuclein aggregation inhibitor [90,100,101] Improves glutamate homeostasis [102,103,104,105] Anti-inflammatory (TLR4/NF-κB pathway) [104] Reduces levodopa side effects [102] Promotes neurogenesis [106,107]	ALS [108,109] Bipolar disorder [110] Refractory psychosis [111]	Gastrointestinal, skin and vascular disorders [112]
Geldanamycin [113]	Antibiotic and a potent antitumor compound	Increases chaperone activity [90,114]	N.A.	Gastrointestinal, hepatic, and eye disorders [115]
Amphotericin B [116]	Fungicide	Reduces prion aggregation [117]	N.A.	Nephrotoxicity, anemia, and cardiomyopathy [118]

## Abbreviations

AD	Alzheimer’s disease
ALS	Amyotrophic lateral sclerosis
Aβ	β-amyloid
BBB	Blood–brain barrier
CI	Cerebral ischemia
CNS	Central nervous system
DAMP	Danger-associated molecular pattern
FQ	Fluoroquinolone
HD	Huntington’s disease
HTT	Huntingtin
IL	Interleukin
KGDH	Ketoglutarate dehydrogenase
LB	Lewis bodies
LPS	Lipopolysaccharide
MELAS	Mitochondrial encephalopathy, lactic acidosis, and stroke-like episodes
mtDNA	Mitochondrial DNA
MVD	Mitochondria-derived vesicles
TOR	Main target of rapamycin
NBIA	Neurodegeneration with brain iron accumulation
NLRP3	NLR family pyrin domain containing 3
NFT	Neurofibrillary tangles
PD	Parkinson’s disease
PDH	Pyruvate dehydrogenase
PrP	Prion protein
PrPC	Cellular prion protein
ROS	Reactive oxygen species
SNpc	Substantia nigra pars compacta
TLR	Toll-like receptor
TNF-α	Tumor necrosis factor-α
UPR^mt^	Mitochondrial unfolded protein response
